# Determination and Commitment of Mechanosensory Hair Cells

**DOI:** 10.1100/tsw.2002.177

**Published:** 2002-04-20

**Authors:** Matthew W. Kelley

**Affiliations:** Section on Developmental Neuroscience, National Institute on Deafness and Other Communication Disorders, National Institutes of Health, 5 Research Court, Room 2B-44, Rockville, MD 20850, USA

**Keywords:** cochlea, auditory system, Math1, Notch1, development

## Abstract

Sound and movement are perceived through the vibration of modified ciliary bundles located on the apical surfaces of specialized mechanosensory hair cells. These hair cells derive from specific regions of the otocyst that become determined to develop initially as sensory epithelia and ultimately as either hair cells or supporting cells. The number of hair cells in an individual vertebrate is surprisingly small and the ability to replace these cells varies among different classes. The molecular and cellular factors that specify hair cell identity are not known, but the results of recent experiments have begun to identify some of the signaling pathways that play important roles in hair cell development. This review will describe recent findings related to the factors that influence the final choice of a progenitor cell to develop as a hair cell and discuss their implications for the overall development of the auditory and vestibular systems.

## INTRODUCTION

In vertebrates, the sensations of hearing and balance are mediated through specialized mechanoreceptors referred to as *hair cells.* The name *hair cell* refers to the presence of a compact bundle of actin-based stereocilia located on the apical surface. Movements of this stereociliary bundle lead to changes in cellular ion permeability that are perceived as sound or motion[1]. The total number of hair cells in a normal man or woman is approximately 30,000 per ear, making these cells relatively rare by comparison with other primary sensory cells, such as the roughly 110 million photoreceptors that populate each human retina[2]. However, in a manner similar to photoreceptors and other types of neurons, the potential for hair cell regeneration, at least in mammals, is extremely limited[3,4,5,6,7,8,9]. These results suggest that the appropriate specification of these cells represents an important step in the formation of auditory and vestibular sensory epithelia. The cellular, molecular, and genetic factors that specifically regulate the determination and commitment of progenitor cells as hair cells are still poorly understood. Nevertheless, studies conducted over the last 5 to 10 years have provided significant insights regarding some of the pathways and molecules that play a role in the formation of these exquisite mechanosensors.

## DEFINITIONS

In order to discuss the development of a specific cell type, it will be necessary to define the different types of cells that play a role in the formation of that cell. Therefore, for the purposes of this article, the following terms will be used to define cell types at specific phases of development:

- **Mitotic cell:** A mitotically active cell located within a developing or mature hair cell epithelia. This cell is assumed to be uncommitted in terms of final cell fate.
- **Prosensory cell:** A postmitotic, uncommitted cell that has the potential to develop as any cell type within a hair cell epithelium, including a hair cell or any type of supporting cell.
- **Precursor cell:** A cell that is committed to develop as a specific cell type but that may not be identifiable based on specific molecular, immunocytochemical, or morphological criteria.
- **Mechanosensory hair cell:** A differentiated sensory cell that has a stereociliary bundle and that generates synaptic activity in response to mechanical stimuli.
- **Supporting cell:** Any cell within a hair cell sensory epithelium that is not a hair cell. These can include highly specialized cells such as inner and outer pillar cells from the organ of Corti, and morphologically indistinct cells such as the supporting cells that are present in the vestibular epithelia of all vertebrate classes.

## DEVELOPMENT OF HAIR CELL SENSORY EPITHELIA

In reptiles, birds, and mammals, hair cell sensory epithelia arise from specific regions of the embryonic otocyst[10,11,12,13]. The otocyst is also the primary source of sensory epithelia in fish and amphibians; however, hair cells located within the lateral line neuromasts derive from multiple lateral line placodes that arise near the developing otocyst[14]. The factors that control the positioning of individual sensory epithelia within the otocyst are not completely understood but appear to involve signals from both the adjacent neural tube and underlying mesenchyme[10,15].

One of the most striking aspects of all mature hair cell sensory epithelia is the existence of a cellular mosaic composed of hair cells and supporting cells[16,17]. Hair cells are located in the most apical strata of the epithelium and their basal and lateral surfaces are surrounded by between 4 and 12 supporting cells[18,19]. As a result, hair cells do not normally contact other hair cells or the basement membrane; however, as will be discussed below, a few examples of contacts between hair cells have been described. In contrast, since most supporting cells make contact with both the lumenal and basal surfaces of the epithelium, the lateral surfaces of supporting cells commonly contact other supporting cells as well as hair cells.

The morphological development of this cellular mosaic has been studied extensively and will be summarized here. Prior to differentiation, developing sensory epithelia are comprised of epithelial cells arranged in a characteristic hexagonal packing pattern[20,21] (Kelley, unpublished observations). At this point in development, most sensory epithelia contain a combination of actively proliferating mitotic cells and postmitotic prosensory cells. Based on morphological criteria, the first cells that can be identified within the epithelia are developing hair cells. These cells can be identified based on changes in the size of cell nuclei and in cytoplasmic staining characteristics[22,23,24,25,26]. In addition, several immunocytochemical markers have been demonstrated to selectively label developing hair cells. These include myosin VI, myosin VIIa, and calretinin in mammals[27,28,29]; β-tubulin, calmodulin, and hair cell antigen (HCA) in chicks[30,31,32]; and acetylated tubulin and *pax2.1* in zebrafish[33]. In general, differentiating hair cells appear to develop in a relatively regularly spaced pattern in which each hair cell is separated from neighboring hair cells by one or more prosensory cells, suggesting that cell-cell interactions may play a role in the initial determination of individual cells as hair cells[18,34]. There have been some reports of contacts between hair cells during early development[19,35,36] and in mature epithelia[37], but it is not clear whether these contacts existed during the period of cellular commitment or whether limited contacts between cells would be sufficient to influence determination.

As development continues, prosensory cells surrounding each hair cell presumably become specified and differentiate as supporting cells. But it should be noted that the degree of specialization of supporting cells varies widely among hair cell epithelia. In the mammalian organ of Corti, supporting cells develop as highly specialized cell types, including inner and outer pillar cells and Deiter's cells. In contrast, supporting cells in virtually all other hair cell sensory epithelia remain morphologically indistinct and, for the most part, homogenous. As a result, the differences between the supporting cells that are present in most mature sensory epithelia and the prosensory cells that gave rise to those epithelia may be limited.

As discussed, the number of supporting cells contacting each hair cell can range from 4 to 12. The factors that determine the number of supporting cells surrounding each hair cell are unknown. In epithelia in which each hair cell is surrounded by six supporting cells, it seems reasonable to assume that the hexagonal packing pattern of the prosensory cells is simply maintained and that all the progenitor cells that do not develop as hair cells go on to develop as supporting cells (Fig. 1A). However, it is less clear how the number of supporting cells surrounding each hair cell is regulated in epithelia in which the number of supporting cells surrounding each hair cell is less than or greater than six. For instance, in the sensory epithelia of the mammalian cochlea, only four supporting cells surround each hair cell (Fig. 1B)[17]. This pattern suggests that there has been some cellular rearrangement or cell loss during development; however, the specific mechanisms involved have not been determined. Similarly, in one region of the chicken basilar papilla, the number of supporting cells surrounding each hair cell increases from 6 to 12[19]. In this case, the increase appears to be the result of both cellular rearrangement and ongoing mitotic proliferation leading to the production of new supporting cells in that region.

## REGENERATION OF HAIR CELLS

In addition to studies that have examined the embryonic development of hair cells, a significant amount of data regarding hair cell commitment has been obtained from studies of hair cell regeneration. Prior to the late 1970s, it was widely assumed that hair cells were only produced during the embryonic period. However, subsequent studies demonstrated that the potential for significant addition and/or regeneration of hair cells exists in all vertebrate classes except mammals[38,39,40,41,42]. Mammals may possess the ability to regenerate a limited number of hair cells in vestibular sensory epithelia[43,44,45], but hair cell regeneration has never been conclusively demonstrated to occur within the mammalian cochlea.

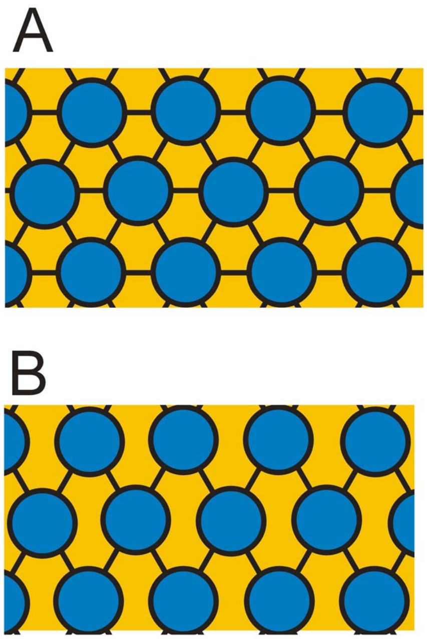

**FIGURE 1.** Sensory hair cell mosaics. All hair cells exist within cellular mosaics of sensory hair cells and nonsensory supporting cells. Diagrams depict the interactions between hair cells and supporting cells at the apical surface of the epithelium. (A) In most cases each hair cell (blue) is contacted by between five and seven supporting cells (gold). (B) However, in the mammalian cochlea, the number of supporting cells contacting each hair cell is limited to four.

In-depth studies of the changes that occur within hair cell epithelia during a regenerative response, in particular in the basilar papilla of the chick, have demonstrated interesting similarities and differences in the formation of hair cells between embryonic and regenerating systems. In response to an insult that leads to hair cell loss, supporting cells located in the region of the loss re-enter the cell cycle and pass through one or more rounds of proliferation[40,41,46,47,48]. Some of the resulting daughter cells go on to differentiate as regenerated hair cells, while others differentiate as supporting cells. Comparisons of the morphological development of hair cells suggest that embryonic and regenerative differentiation do not differ significantly[32,49]. As discussed, most supporting cells, including those within the basilar papilla, are not morphologically distinct from prosensory cells. Moreover, no significant differences between the two cell types have been demonstrated at the molecular level. Therefore, it seems possible that supporting cells could, in fact, also be prosensory cells. One argument against this hypothesis is the observation that, in most if not all cases, supporting cells do not appear to have the potential to change their fate and to develop as hair cells. Rather, at least a single round of mitosis is required prior to the differentiation of a newly postmitotic supporting cell daughter as a hair cell. As will be discussed below, there is some data suggesting that a limited number of supporting cells may be able to undergo a direct change in fate[50,51,52,53,54,55,56], but recent data suggests that these cells may be injured hair cells in the process of recovery rather than supporting cells in the process of a change in fate[57,58].

## TIMING OF HAIR CELL DETERMINATION

A key step in understanding the factors that play a role in the determination of the hair cell fate is to identify the time period in which cells become committed to develop as hair cells. Classically, commitment to a specific cell fate has been demonstrated using a combination of cell ablation and cell transplantation studies. In particular, transplantation studies have been used to determine the timing of the acquisition of a fully autonomous level of cell specification. Unfortunately, the small number of prosensory cells in the otocyst and the relative inaccessibility of the inner ear have served as major impediments to the use of these types of studies for the determination of hair cell commitment. However, it is possible to infer the timing of hair cell determination based on the results of retroviral lineage studies of cell fates, as well as from descriptive studies of the early differentiation of cells as hair cells.

If mitotic cells within the developing otocyst become determined to develop as hair cells prior to terminal mitosis, then individual cells that give rise to clones comprised entirely of hair cells should be present at some point in development. However, the results of retroviral lineage studies in the embryonic chick otocyst suggest that the composition of all clones, regardless of the size of the clone, is random[59]. Similarly, analysis of the fates of the two daughter cells that arise from a single terminal mitosis indicates that the fates of these cells are variable. These results are consistent with the hypothesis that prosensory cells do not become determined as hair cells until during or after the preceding terminal mitosis[59].

During both development and regeneration, mitotically active cells undergo active vertical internuclear migration, with cytokinesis occurring at the lumenal surface of the epithelium (Fig. 2)[60,61,62,63]. As a result, the daughter cells that will ultimately develop as both hair cells and supporting cells are initially located in the most apical region of the epithelium. Daughter cells that will develop as supporting cells must undergo further vertical nuclear migration and reestablish contact with the basement membrane. In contrast, daughter cells that will develop as hair cells are already close to their final position within the sensory epithelium. Therefore, if individual cells became determined as hair cells during or soon after terminal mitosis, as has been suggested for developing cortical neurons[6,64,65], then there would be no need for those cells to undergo further internuclear migration. In fact, the results of a recent study of hair cell regeneration in the chick basilar papilla (cochlea) demonstrated the expression of hair cell–specific markers in BrDU-labeled cells within 14 h of BrDU addition[63]. At this time point, developing hair cells are located near the lumenal surface of the epithelium and appear rounded. However, as these cells continue to develop, they elongate and extend a basal process that may or may not re-establish contact with the basement membrane. At the same time, the nuclei of these cells move downward within the epithelium. Finally, the cells once again become rounded, retract their basal processes, and move their nuclei upward within the epithelium to assume a position consistent with mature hair cells. Similar cellular morphologies have been observed in the embryonic avian basilar papilla[32,66,67], suggesting that all developing hair cells may follow the same developmental pattern. The basis for these morphogenetic changes is unclear. If a cell has already become committed to develop as a hair cell, why does the cell then undergo cellular extension and nuclear migration only to ultimately return to its original morphology? Moreover, is there any functional significance to the extension of a cytoplasmic tail that may re-establish contact with the basement membrane? During the differentiation of skin epithelia, contact with the basement membrane acts as an inhibitory signal that prevents cellular differentiation[68,69,70,71]; since developing hair cells may re-establish contact with the basement membrane, this suggests that similar inhibitory interactions may not occur in this system. These results suggest the possibility that expression of some early hair cell–specific markers may not be indicative of the commitment of prosensory cells to the hair cell fate and that instead individual prosensory cells may require additional signals prior to commitment. Alternatively, prosensory cells may become committed to a hair cell fate soon after terminal mitosis, but subsequent morphogenetic movements may be required for the appropriate differentiation of these cells.

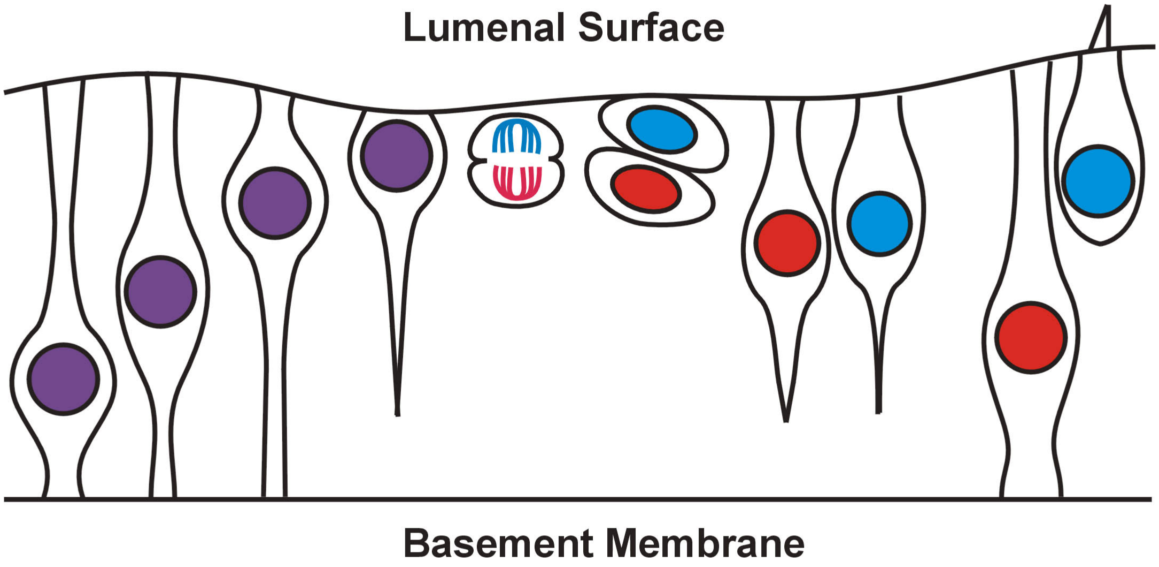

**FIGURE 2.** Internuclear migration and hair cell differentiation. Time is expressed from left to right. Within a developing hair cell sensory epithelium, an individual epithelial cell spans the distance between the basement membrane and the lumenal surface. During interphase, the nucleus of the cell (purple) is located close to the basement membrane. As the cell progresses through the cell cycle, the nucleus migrates towards the lumenal surface. The basal process of the cell is retracted prior to mitosis. The plane of cleavage can be variable but is depicted as parallel to the lumenal surface. After mitosis, each of the daughter cells (red and blue nuclei) may develop as either a hair cell or a supporting cell. The developing supporting cell (red) extends a cytoplasmic process that eventually makes contact with the basement membrane. The developing hair cell (blue) extends a slender process that may or may not contact the basement membrane (see text for details). As differentiation continues this process is retracted and the cell assumes a flask-like shape.

The results described above suggest that, in a manner similar to most developing neuronal systems, the initial stages of hair cell determination probably occur during or fairly soon after terminal mitosis. As a result, in developing vestibular sensory epithelia and during regeneration, the pattern of hair cell differentiation parallels, with a slight delay, the pattern of terminal mitosis, suggesting that determination occurs during this relatively brief delay[29,72]. There are, however, two striking exceptions to this general pattern. In the embryonic mammalian cochlea, terminal mitosis occurs in an apical-to-basal gradient[73], while hair cell differentiation proceeds in a basal-to-apical gradient[19,23,25,26,74]. As a result, for cells located in the apex of the cochlea, the time between terminal mitosis and the initial stages of hair cell differentiation can be as long as 96 h. Similarly, in the embryonic avian basilar papilla, terminal mitoses are first observed on E6 in a medio-lateral band that extends along the proximo-distal axis[75]. During the next 3 days, additional terminal mitoses occur in a gradient that progresses towards both the neural and abneural edges of the epithelium. During the same time period, hair cell differentiation occurs in a distal-to-proximal gradient that initiates at the distal end of the basilar papilla[76,77,78,79]. Therefore, for cells located in the medio-proximal region of the papilla, the time between terminal mitosis and differentiation may be as long as 72 h. These results suggest that either the determination or differentiation of some progenitor cells within the epithelium may be delayed by several days. Therefore, while the determination of most cells as hair cells appears to occur relatively soon after terminal mitosis, there appear to be some progenitor cells that delay either determination or differentiation by a significant period of time. While it is not clear whether this delay represents a delay in determination or differentiation, the results of laser ablation experiments that will be described in a following section suggest that the delay occurs in determination rather than differentiation.

## SYMMETRIC VS. ASYMMETRIC CELLULAR DIVISIONS

Events that occur during terminal mitosis can play a key role in the determination of the cellular phenotypes of the resulting daughter cells[80,81,82,83]. In particular, the asymmetric localization of cellular determination factors such as Numb and Prospero to opposite poles of a cell prior to cytokinesis has been demonstrated to direct distinct cellular fates in the daughter cells that arise from that division. The frequency of symmetric and asymmetric divisions during hair cell development or regeneration has not been examined extensively. Nevertheless, existing data suggest that asymmetric divisions may play a role in the determination of cells as hair cells. Analysis of a limited number (8) of two-cell clones from a retroviral lineage analysis study of the chicken otocyst indicated the potential for both hair cell:supporting cell (62%) and supporting cell:supporting cell (38%) clones[59]. Hair cell:hair cell clones were not observed. Similarly, time-lapse based lineage analysis of hair cell regeneration in lateral line neuromasts of axolotls indicated that most terminal mitoses lead to the formation of one hair cell and one supporting cell[50]. In addition, several studies of the fates of isolated pairs of closely associated postmitotic cells in the chick utricle indicated that pairs are usually comprised of a single hair cell and a single supporting cell[49,63,84,85]. Finally, Numb, a cytoplasmic protein that is asymmetrically localized within a cell prior to a mitotic division that leads to daughter cells with different fates, has been localized to hair cells by immunocytochemistry[86]. These results strongly suggest that asymmetric divisions may play a key role in the determination of cells as hair cells during embryonic development.

In contrast with embryonic development, an analysis of the fates of closely related pairs of BrDU-labeled cells in regenerating chicken basilar papilla indicated that under these conditions as many as 33% of all terminal mitotic divisions may lead to symmetric divisions in which both daughters develop as hair cells[49,63]. Therefore, while the existing data support a role for asymmetric divisions in the determination of hair cells during embryonic development, the data from a regenerating system suggest that asymmetric divisions are not required for hair cell determination. Clearly, further studies are required to fully understand the role of asymmetric division in hair cell development.

## EFFECTS OF CELL ABLATIONS

The observation that hair cells are the first cell types to differentiate within the developing sensory epithelia and that these cells appear to develop in a regularly spaced pattern led to the hypothesis that the hair cell fate might represent the primary cell fate within the epithelium and that, once a cell became committed to develop as a hair cell, that cell might produce an inhibitory signal that prevented surrounding prosensory cells from differentiating as hair cells[18,34]. This hypothesis assumed that the prosensory cells within the developing epithelium represent an equivalence group in which any cell can develop as either a hair cell or a supporting cell.

To test the hypothesis that developing hair cells generate an inhibitory signal that prevents surrounding cells from developing as hair cells, newly developing hair cells in the embryonic mouse cochlea were ablated by laser microbeam irradiation[87]. Analysis of the responses of cells located adjacent to ablated hair cells indicated that in response to the loss of a developing hair cell, an existing prosensory cell located adjacent to that cell would develop as a hair cell. It is important to note that the fates that these prosensory cells would have assumed in the absence of hair cell ablations cannot be determined. However, the position of these cells within the epithelium, along with the results of control experiments, strongly suggest that these cells would have developed as supporting cells[87]. Therefore, these results indicate that developing hair cells produce an inhibitory signal that prevents neighboring cells from developing as hair cells and that the disruption of this signal probably leads to a change in the fate of one of the surrounding prosensory cells.

A second series of experiments from this same study determined whether the delay in the differentiation of progenitor cells located in the apical region of the cochlea represents a delay in commitment or differentiation. Laser ablations made in different regions of the cochlea and at different developmental time points indicated that the ability of cells to changes fates in response to laser ablations was maintained for longer periods of time in cells located in the apical region of the cochlea. Since these cells represent the first cells to become postmitotic, this suggested that there is a delay in the commitment rather than the differentiation of prosensory cells in the apical region of the cochlea. The results also demonstrated that within the mammalian cochlea the ability of cells to change developmental fate in response to changes in the environment is limited to a finite time period during embryonic development.

Nevertheless, this loss of cellular plasticity may be unique to the mammalian cochlea. As discussed, hair cell regeneration has been demonstrated in a broad spectrum of vertebrate classes and in all hair cell sensory epithelia except the mammalian cochlea. A majority of regenerated hair cells appear to arise from mitotically active precursor cells, but the results of several studies have suggested that some regenerated hair cells may arise through the direct conversion of existing supporting cells into hair cells (referred to as *transdifferentiation* or *conversion*)[50,51,52,53,54,55,56]. The significance of the concept of supporting cell conversion has recently been questioned as a result of observations that indicate that some treatments that were thought to lead to hair cell death instead only lead to hair cell injury[57,58]. In response to this injury, a hair cell will shed its apical half and the remaining portion of the cell will lose its contact with the lumen. As these cells recover, they pass through a morphological phase that may appear similar to a converting hair cell. As a result, it may be the case that many of the reported instances of converting supporting cells are actually recovering hair cells. However, the observation of even a limited number of converting supporting cells suggests that some of the cells that are classified as supporting cells may also be considered to be prosensory and capable of a change in fate in response to environmental cues.

In summary, the results of laser ablation studies have demonstrated that the removal of a hair cell can lead to a change in the fate of one of the cells located adjacent to the ablated cell, suggesting that hair cells produce an inhibitory signal that prevents neighboring cells from developing as hair cells. These results are also consistent with the hypothesis that the hair cell fate represents the primary cell fate for the epithelial equivalence group that gives rise to hair cell sensory epithelia.

## *MATH1* ACTS AS A PRONEURAL (PROSENSORY) GENE FOR THE HAIR CELL FATE

The results described in the previous section suggest that the hair cell fate should be specified through the expression of a proneural, or, more appropriately, prosensory gene. Briefly, this gene should be initially upregulated within all of the cells of the equivalence group and expression of that gene should be sufficient to commit cells to a hair cell fate. However, as development continues, the overall expression of that gene should be downregulated in cells that will not develop as hair cells. Recent results have demonstrated that *Math1,* the mammalian homologue of the *Drosophila* basic helix-loop-helix gene *atonal*[88], acts as a prosensory gene for the hair cell fate. *Math1* is initially expressed broadly in the developing vestibular and auditory sensory epithelia of the mouse as well as in the developing neuromasts of zebrafish[89,90,91]. In the mouse, the onset of *Math1* expression roughly correlates with the timing of terminal mitosis; however, specific experiments have not been conducted[89,90]. As development continues, *Math1* expression persists in cells that will develop as hair cells but is downregulated in cells that will develop as supporting cells[89,90]. Deletion of *Math1* in mice results in the complete absence of hair cells[89], and overexpression of *Math1* is sufficient to induce nonsensory epithelial cells within the cochlear duct to develop as hair cells[92]. Interestingly, a second proneural gene, NeuroD[93], is also expressed in developing hair cells; however, development of hair cells is normal in NeuroD mutant mice[94], indicating that this molecule is not required for hair cell development.

## THE NOTCH PATHWAY REGULATES THE NUMBER OF CELLS THAT DEVELOP AS HAIR CELLS

As discussed, developing hair cells apparently generate a signal that inhibits neighboring cells from developing as hair cells. Similar inhibitory interactions between adjacent cells have been described during the development of other organisms, such as *Drosophila* and *C. elegans,* and in most cases the basis for this inhibitory interaction is signaling through the Notch pathway[95,96,97,98,99]. Briefly, a membrane-bound receptor, Notch, is activated by binding of a membrane-bound ligand (Delta, Serrate, or Jagged). Binding of the ligand leads to Notch receptor activation and apparently to the inhibition of differentiation primarily through downregulation of proneural gene expression.

Several different members of the Notch signaling pathway are expressed in the developing auditory system; however, the discussion here will be restricted to the role of Notch signaling in the final determination of cells as hair cells. At the time of *Math1* upregulation, Notch message and protein are expressed throughout developing hair cell sensory epithelia as well[100,101,102,103,104,105]. Soon after the onset of *Math1* expression, individual cells within the *Math1* domain begin to express Notch ligands (*Jagged2* and *Delta1* in mouse[104,105,106]; *Delta1* in chicken[101]; and *DeltaA*, *DeltaB*, and *DeltaD* in zebrafish[33,91,102]). As development continues, cells that express Notch ligands go on to differentiate as hair cells. Cells located adjacent to ligand-expressing cells upregulate Notch target genes such as *HES5*[90] and *HES1*[107,108]. At the same time, *Math1* is downregulated in these cells[90,91]. Deletion of *Jagged2* in mice leads to an overproduction of hair cells in the cochlea[104], as does treatment of cochlear cultures with *Notch1* antisense oligonucleotides[109]. Similarly, disruption of the Notch signaling pathway in zebrafish with a mutation in the *DeltaA* gene[33] or in zebrafish mindbomb mutants also leads to an increase in the number of cells that develop as hair cells[102,110]. In the cases of *Jagged2* deletions and mindbomb mutants, disruption of the Notch signaling pathway results in an increase in the number of cells that maintain expression of *Math1*[91,104]. Finally, overexpression of *HES1* has been shown to be sufficient to inhibit Math1-mediated induction of hair cells[107,108] and the expression of *HES5* in supporting cells is dependent on expression of *Jagged2* in developing hair cells[90].

It is important to note that a comparison of the pattern of expression for *Delta1* in the chicken basilar papilla during embryonic development and adult regeneration demonstrates intriguing differences. As discussed, during embryonic development of the basilar papilla, *Delta1* is apparently expressed only in the subset of prosensory cells that will develop as hair cells[101]. In contrast, during regeneration in the adult basilar papilla, *Delta1* is expressed in approximately equal amounts in the two daughter cells arising from single mitotic divisions[85]. As regeneration continues, *Delta1* is maintained in cells that will develop as hair cells and is downregulated in cells that will develop as supporting cells. These results — along with the observation that during regeneration many mitotic divisions can be symmetric, leading to the generation of two hair cells — suggest that *Delta1* may play different roles during development and regeneration. The observation of restricted expression of *Delta1* and of largely asymmetric divisions suggests that during development *Delta1* is only expressed in a cell that is destined to become a hair cell. On the other hand, during regeneration *Delta1* appears to be expressed prior to the choice of cell fate, suggesting that subsequent interactions play a role in both the regulation of *Delta1* expression and in the final determination of cell fate[85].

In summary, the results of studies on Notch signaling suggest that this pathway plays a role in the regulation of the number of cells that will develop as hair cells. The results also suggest that developing hair cells directly inhibit adjacent cells from developing as hair cells. However, it is important to note that decreases in the number of cells that develop as supporting cells has only been reported in the mindbomb[102,110] and *DeltaA*$^{dx2}$[33] mutants. It is not clear whether this is a result of functional redundancy in mammalian systems or of a secondary induction of supporting cells by differentiating hair cells.

## LUNATIC FRINGE REGULATES THE DEVELOPMENT OF INNER HAIR CELLS

Fringe proteins act to modulate the response of Notch receptors through glycosylation of specific EGF repeats in the extracellular domain of the protein[97,111,112]. In particular, fringe-mediated glycosylation of Notch results in enhanced activation in response to binding by Delta1 but to decreased activation in response to Jagged1 binding[113,114]. Lfng is expressed in progenitor cells located in developing sensory patches throughout the auditory system in both chickens and mice[115,116]. However, lfng expression is progressively downregulated in cells that will develop as hair cells and, in the mammalian cochlea, in cells that will develop as inner pillar cells[115,116,117]. However, cochleae from lfng mutant animals are phenotypically normal[117]. As discussed, deletion of Jagged2 or Notch1 leads to an increase in the number of both inner and outer hair cells in the cochlea[104,109]. Surprisingly, cochleae from animals that are homozygous negative for both Jagged2 and Lfng have an increased number of outer hair cells but a normal complement of inner hair cells[117]. A theoretical explanation for this phenotype that is consistent with the existing data regarding the effects of Lfng has not been presented. However, a preliminary analysis of the phenotype in Jag2/Lfng double mutants suggests that the number of pillar cells in these animals may be increased compared to wild type (Kelley, unpublished observations). This would suggest that specific levels of Notch activation might be required to prevent progenitor cells from developing as pillar cells.

## HOW DO INDIVIDUAL PROSENSORY CELLS BECOME DETERMINED TO DEVELOP AS HAIR CELLS?

Based on the results described above, the following model can be proposed: Developing hair cell sensory epithelia are initially comprised of prosensory cells that have the potential to develop as either hair cells or supporting cells (Fig. 3). Around the time of their terminal mitoses, these cells exit the cell cycle and begin to express the bHLH gene Math1; however, it is not clear whether the onset of Math1 expression occurs prior to or after terminal mitosis. Expression of Math1 initiates a program that is necessary and sufficient for the differentiation of any prosensory cell as a hair cell. Following terminal mitosis, all prosensory cells begin to move away from the lumenal surface and extend cytoplasmic projections towards the basement membrane. Within several hours, a subset of prosensory cells becomes determined to develop as hair cells. At this point the cells begin to express species-specific hair cell markers and movement away from the lumenal surface is reversed. At the same time, these cells begin to express ligands for Notch. Binding of these ligands to Notch receptors on adjacent cells leads to activation of Notch and expression of Notch target genes such as HES5 or HES1, which leads to downregulation of Math1 and diversion from the primary (hair) cell fate. These cells presumably go on to differentiate as supporting cells.

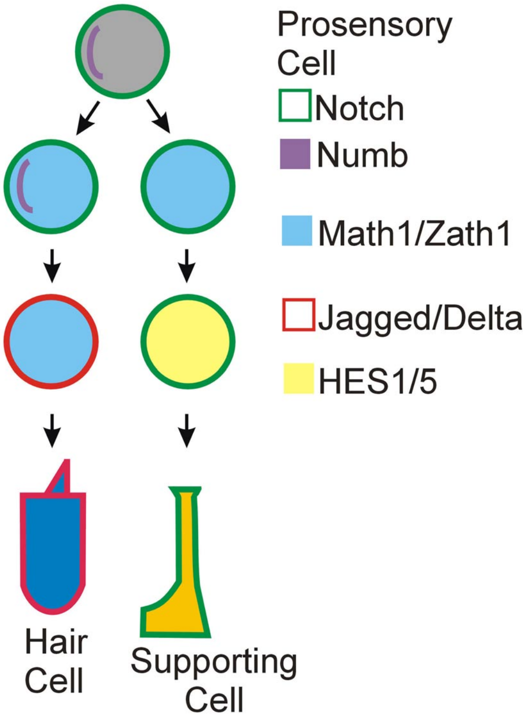

**FIGURE 3.** Summary diagram for the molecular signals that play a role in the determination of cells as hair cells. Initially, an epithelial cell divides to give rise to two prosensory daughter cells. During the final division, numb protein may become restricted to one of the two daughter cells, leading to a relative decrease in the level of Notch activation in that cell. Following terminal mitosis, each of the prosensory daughter cells begins to express an Ath1 protein. Next, the cell that will develop as a hair cell upregulates the expression of one or more Notch ligands, such as Jagged2 or Delta1. The factors that regulate ligand expression have not been determined, but asymmetrical localization of Numb could play a role. Expression of a Notch ligand leads to activation of Notch in adjacent cells. Activated Notch induces a downstream signaling pathway that results in the expression of the inhibitory bHLH proteins HES5 and/or HES1. Expression of HES proteins leads to inhibition and downregulation of Ath1. As a result, these cells are diverted from developing as hair cells and ultimately develop as supporting cells.

The factors that single out individual cells to become determined to develop as hair cells are not known. There is data to suggest that asymmetric distribution of numb during terminal mitosis may bias some cells towards the hair cell fate by inhibiting notch activity[86]. However, the data from regenerating systems suggests that asymmetric divisions are apparently not necessary for hair cell determination[49,63]. Rather, it appears that during hair cell regeneration both daughters from a single mitosis can develop as hair cells. It is not clear why such a difference exists; however, it is possible that asymmetric divisions might play a role in the development of the cellular mosaic. In fact, there have been reports of patterning defects in the cellular mosaic after a regenerative event[118], supporting the hypothesis that asymmetric divisions may play a role in patterning. These results suggest that expression of Math1 is sufficient for the determination of a prosensory cell as a hair cell but that the ultimate commitment of a cell to the hair cell fate may depend on either biochemical or stoichiometric interactions that lead to a decrease in the level of Notch activity relative to surrounding cells.

## SUMMARY

Mechanosensory hair cells exist in specialized sensory epithelia that are comprised of both sensory hair cells and nonsensory supporting cells. Both cell types apparently form from an equivalent group of multipotent prosensory cells. Initially, all cells within the equivalence group express Math1, a protein that has been shown to be necessary and sufficient for the determination of a cell as a hair cell. Subsequent cell-cell interactions lead to the generation of two distinct populations of cells, one of which expresses Math1 and a Notch ligand such as Jagged2 or Delta1 and will develop as hair cells and a second that expresses Notch and Notch targets such as HES1 or HES5. This second population of cells will differentiate as supporting cells. The cellular and molecular factors that lead to the commitment of an individual prosensory cell as a hair cell have not been determined. However, since activation of Notch can lead to the inhibition of Math1 activity and expression, downregulation of Notch is probably a key step. Asymmetric cellular divisions appear to occur during embryonic development and one result of those divisions may be the segregation of the Notch-inhibitory protein Numb to cells that will subsequently develop as hair cells. But asymmetric divisions are apparently not required during hair cell regeneration in the chick, suggesting that the commitment of individual prosensory cells as hair cells can also occur randomly as a result of differential levels of expression of Notch and Notch ligands. The existence of asymmetric divisions during embryonic development of hair cell sensory epithelia suggests that, in this system, asymmetry may play a role in cellular patterning rather than cellular commitment.
